# Treatment of two postoperative endophthalmitis cases due to *Aspergillus flavus *and *Scopulariopsis *spp. with local and systemic antifungal therapy

**DOI:** 10.1186/1471-2334-7-87

**Published:** 2007-07-31

**Authors:** Sayime Aydin, Bulent Ertugrul, Berna Gultekin, Guliz Uyar, Erkin Kir

**Affiliations:** 1 The Hospital of Dumlupinar University, Department of Ophthalmology, Kutahya, Turkey; 2 Adnan Menderes University Medical Faculty, Department of Infectious Diseases and Clinical Microbiology, Aydin, Turkey; 3 Adnan Menderes University Medical Faculty, Department of Microbiology and Clinical Microbiology, Aydin, Turkey; 4 Adnan Menderes University Medical Faculty, Department of Infectious Diseases and Clinical Microbiology, Aydin, Turkey; 5 Adnan Menderes University Medical Faculty, Department of Ophthalmology, Aydin, Turkey

## Abstract

**Background:**

Endophthalmitis is the inflammatory response to invasion of the eye with bacteria or fungi. The incidence of endophthalmitis after cataract surgery varies between 0.072–0.13 percent. Treatment of endophthalmitis with fungal etiology is difficult.

**Case Presentation:**

**Case 1: **A 71-year old male diabetic patient developed postoperative endophthalmitis due to *Aspergillus flavus*. The patient was treated with topical amphotericin B ophthalmic solution, intravenous (IV) liposomal amphotericin-B and caspofungin following vitrectomy.

**Case 2: **A 72-year old male cachectic patient developed postoperative endophthalmitis due to *Scopulariopsis *spp. The patient was treated with topical and IV voriconazole and caspofungin.

**Conclusion:**

*Aspergillus *spp. are responsible of postoperative fungal endophthalmitis. Endophthalmitis caused by *Scopulariopsis *spp. is a very rare condition. The two cases were successfully treated with local and systemic antifungal therapy.

## Background

Endophthalmitis is an intraocular infection caused by bacteria or fungi. Eventhough it is a rare condition, therapeutic options are limited and it affects the vision seriously. The incidence of endophthalmitis after cataract surgery varies between 0.072%–0.13% as reported in publications of the past 10 years [1]. In 83% of postoperative endophthalmitis cases, the infectious agents are bacteria, especially gram positive bacteria. However, few studies have been based on collected microbiology data regarding fungi. In these series the rate of fungal endophthalmitis varies between 8.6–18.6% [2,3]. This aspect is also lacking in another large prospective series[4]. *Candida albicans *and *Aspergillus *spp. are the most frequently isolated organisms in fungal cases [2,5]. Treatment of endophthalmitis with fungal etiology is difficult. Systemically or intra-vitreally administered amphotericin-B is the most commonly used drug in the treatment of fungal endophthalmitis. It has been reported that it can also be given in the anterior chamber [6]. In recent years, new anti-fungal agents such as caspofungin and voriconazole have been developed as an alternative to amphotericin-B, and successful results were reported [7-10].

The present article discusses the outcomes of two cases of endophthalmitis due to *A. flavus *and *Scopulariopsis *spp. following phacoemulsification and intraocular lens (IOL) implantation.

## Case Presentation

### Case 1

**Figure 1:**
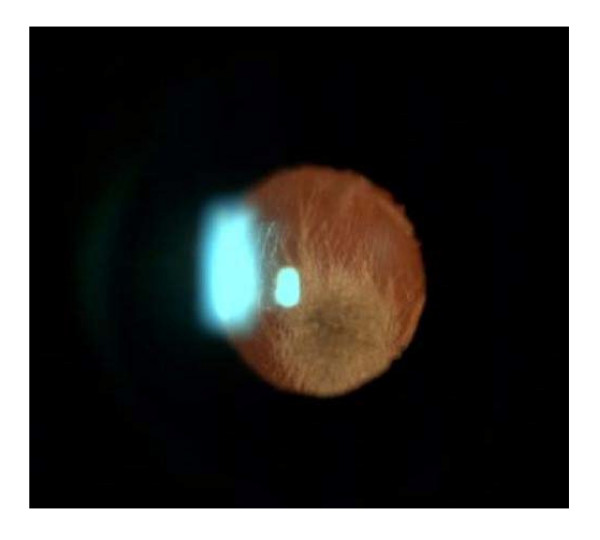
The biomicroscopic image of endophthalmitis due to *Aspergillus flavus *in case 1.

A 71-years old male, diabetic patient underwent phacoemulsification and IOL implantation for right-sided senile cataract. He underwent an operation due to colon cancer 4 years before cataract surgery and received chemotherapy for one year. He had controlled diabetes without signs of diabetic end organ diseases at the time of cataract surgery. Visual acuity was 0.7 preoperatively and achieved 1.0 at postoperative 2^nd ^week. Topical steroid and antibiotic drops were prescribed postoperatively. Medication was continued for one month. At postoperative third month, patient presented visual acuity decrease and redness in the operated eye. Visual acuity was counting finger at one meter. Slit lamp biomicroscopic examination showed ciliary injection, anterior chamber reaction and cotton fiber appearance between the IOL and posterior capsule (Figure 1). This was not noticed in previous follow-ups. Therefore possibility of a foreign body such as cotton was excluded. The appearance was similar to mould hyphae. Endophthalmitis was suspected and anterior chamber fluid and vitreous examples were cultured preoperatively. Amphotericin-B (5 μg/0.1 cc) and cefuroxime (1 mg/0.1 cc) were administered into the anterior chamber while amphotericin-B (10 μg/0,1 cc), amikacin (0.4 mg/0.1 cc) and vancomycin (1 mg/0.1 cc) were given intravitreally. Despite this treatment, biomicroscopic and clinical appearance continued to deteriorate and IOL and capsular remnants were removed with anterior vitrectomy. Anterior chamber fluid, and vitreous examples were cultured perioperatively, IOL was also cultured. Amphotericin-B (5 μg/0.1 cc) and cefuroxime (1 mg/0.1 cc) were administered into the anterior chamber. Topical amphotericin B (0.1 mg/cc) ophthalmic solution and intravenous (IV) liposomal amphotericin-B (Ambisome^®^) (3 mg/kg/day) were started. *Aspergillus flavus *was isolated from the cultures. Dense fibrinoid reaction developed around the pupil and in the vitreous during the follow-up period. Pars plana vitrectomy and silicon oil injection were performed. Intraocular triamcinolone (0.4 mg/0.1 cc) and amphotericin-B (2.5 μg/0.1 cc) injection performed. In-vitro susceptibility with E test revealed that the pathogen's MIC for amphotericin-B was 4 μg/ml. Consequently, IV caspofungin (Cansidas^®^), with a loading dose of 70 mg for one day followed by a maintenance dose of 50 mg/day for the following days, was added to IV liposomal amphotericin-B. In the second week of the treatment, nephrotoxicity due to systemically used liposomal amphotericin-B occurred. Therefore the treatment was continued with caspofungin only. Following the 6-weeks treatment, corrected visual acuity was 0.5 (based on logMAR chart) and caspofungin treatment was terminated. No signs or symptoms of endophthalmitis were observed during the 6-months follow-up.

### Case 2

A 72-year old male patient with phthisical right eye had phacoemulsification and IOL implantation for senile cataract of the left eye. The patient, weighing only 36 kg, was cachectic secondary to eating problems (nausea and vomiting) due to gastrectomy operation. He underwent this operation five years ago. He had received oral supplementation (with high protein content) before the cataract surgery. Fundus examination showed "dry type" age related macular degeneration. Preoperative visual acuity was 0.1 (based on Snellen chart) and achieved 0.5 (based on Snellen chart) after an uneventful phacoemulsification surgery. Topical steroid and ciprofloxacin treatment was started postoperatively. These medications were gradually reduced over a period of one month and stopped. The patient was referred to us for uveitis at postoperative third month. He was suffered from decreased vision and pain. Topical steroid drops had been prescribed. Visual acuity limited to hand motion and biomicroscopic examination showed inflammatory reaction in the anterior chamber. There was a little improvement of the symptoms with steroid treatment. However, complaints were eventually intensified. Fibrinoid inflammatory reaction and membrane formation were noted around the pupil and the anterior chamber. Considering endophthalmitis, anterior chamber lavage was performed. Necrosis of and defect in iris were observed beneath the membrane. Samples of anterior chamber fluid were cultured. Vitreous was clean. Vancomycin (1 mg/0.1 cc) and amphotericin-B (5 μg/0.1 cc) were administered into the anterior chamber. The patient was treated empirically with topical fortified gentamicin and vancomycin ophthalmic solutions, oral ciprofloxacin (750 mg, b.i.d), intravenous (IV) vancomycin (500 mg, q.i.d) and meropenem (1 g, t.i.d). Due to suspicious fungal growth in culture, IV liposomal amphotericin-B (3 mg/kg/day) was started. Secondary to recurrence of dense fibrinoid inflammatory reaction, IOL extraction, vitrectomy and silicon oil injection were performed. Cornea was also edematous. Voriconazole (2.5 μg/0.1 cc) was given into the silicon-filled vitreous space and amphotericin-B (5 μg/0.1 cc) into the anterior chamber. Culture results yielded *Scopulariopsis *spp. In vitro susceptibility with E test revealed that the infecting organism had a MIC of >32 μg/ml for amphotericin-B. MIC for voriconazole (VFEND^®^-Pfizer) was found 8 μg/ml with susceptibility test using dilution method and MIC for caspofungin (Cancidas^®^-MERCK SHARP & DOHME) was 4 μg/ml. Henceforth, IV amphotericin-B was stopped and IV voriconazole (4 mg/kg, b.i.d) and caspofungin (50 mg/day) were started. Voriconazole (2.5 μg/0.1 cc) was injected into the silicon-filled vitreous space. Topical voriconazole (0.01 mg/cc) ophthalmic solution was started. As the hepatic enzymes elevated on the tenth day of the treatment, IV voriconazole was stopped and topical voriconazole and IV caspofungin were administered for a course of 8-weeks. Visual acuity improved to counting finger from 3 meters at the postoperative 2^nd ^month. Fungal endophthalmitis did not recur during the 4 months follow-up period. Corneal edema was still present and visual acuity remained unchanged.

## Conclusion

Endophthalmitis secondary to cataract surgery is a rare but serious condition that affects vision. Previous studies have shown that fungi are responsible of 8.6–18.6% of all culture positive postoperative endophthalmitis [2,3]. Endogenous fungal endophthalmitis usually occurs secondary to dissemination of organisms from a distant focus to the eye via blood and 2–15% of all endophthalmitis cases are estimated to occur in this way [11-13]. On the other hand, exogenous endophthalmitis occurs following eye surgery, penetrating traumas or corneal ulcerations. Even though both types are rare, diseases with abnormalities in immune system (diabetes, renal insufficiency, malignancies and AIDS), intravenous drug addiction, endocarditis, recent major surgery, organ transplantation, corticosteroid and cytotoxic drug use, prolonged antibiotic use, long term intravenous catheterization, genito-urinary and dental interventions increase the risk of endophthalmitis [12-15]. Postoperative fungal endophthalmitis is generally secondary to contaminated intraocular irrigation fluids, air conditioning systems and cluster infections during construction activities in hospitals [16-18].

*Candida albicans *and *Aspergillus *spp. are the most frequently isolated organisms in fungal endophthalmitis [2,16,19,20]. *Candida *spp. are isolated in the majority of endogenous endophthalmitis whereas *Aspergillus *spp. are responsible of postoperative fungal endophthalmitis [3,5]. Aspergillus endophthalmitis is usually caused by *A. fumigatus *and *A. flavus *[21,22]. With the exception of a single case, there is no information on endophthalmitis due to *Scopulariopsis *spp. in the literature [23].

Previous studies have shown that exogenous fungal endophthalmitis has a long latent period, lasting for weeks, even months after intraocular inoculation, and the mean latent period was 7 weeks among these cases [24]. In a study of Narang and colleagues [16] fungal endophthalmitis was noted even 90–210 days after the operation. First line of immunological defense against these fungi is the macrophages which engulf conidia. Hyphae are eliminated mainly by neutrophils. Disorders of neutrophil function play the major role in the development of infection by these pathogens in invasive aspergillosis models. Especially in diabetic patients, poor glycemic control is known to impair immune systems [25,26]. Same effect is observed after steroid; steroids facilitate the development of infection by impairing the defense mechanisms against the fungus exogenously acquired. We re-assessed our cases in the light of this information but failed to identify another endogenous focus following physical examination and analyses. The first case had type II diabetes that was not under control especially in the last months. The second case had excessive cachexia and biochemical analyses revealed excessive hypoproteinemia and hypoalbuminemia. Ophthalmic steroid solutions were used in both patients to suppress the local post operative inflammation. However, these solutions may impair the immune defense.

There are very few agents available for the treatment of fungal endophthalmitis. Moreover, inability to routinely test the sensitivity of the fungal pathogens under laboratory conditions presents a challenge in deciding on the treatment. Until recently, first choice in medical treatment of fungal endophthalmitis is systemic and intravitreal amphotericin-B [27-31]. However, development of resistance in fungal pathogens and concerns of focal retinal necrosis that might occur even with low doses of amphotericin-B have prompted new therapeutic alternatives to be developed. We are lacking sufficient clinical data on the efficacy of combination antifungal therapy. The results of in-vitro studies and animal models suggest that combination antifungal therapy may have additive activity against fungal infections. In some studies, combination of caspofungin and amphotericin-B was shown to have synergistic or additive against *Aspergillus *spp. and *Fusarium *spp. (marked decrease in MIC of both) [32]. As caspofungin inhibits fungal cell wall synthesis, penetration of amphotericin B through the cell membrane is facilitated. Combination antifungal therapy is an alternative approach in infections with multidrug resistant fungi and/or infections that do not respond to standard therapy. [33]. In our first case, antifungal susceptibility test revealed a MIC of 4 μg/ml for amphotericin-B. Therefore caspofungin was added to the treatment. Acute renal failure developed on the second week of treatment which required termination of amphotericin-B. Treatment was continued with caspofungin to complete the 6-weeks course. In the second case, *Scopulariopsis *spp. was isolated with a serious resistance pattern. *Scopulariopsis *spp is known for its resistance to amphotericin-B. On the other hand, we are lacking sufficient data regarding intraocular penetration of caspofungin and its efficacy in ophthalmic infections. However, there are enough studies on voriconazole. Marangon and colleagues [8] carried out a study to determine in-vitro susceptibility of pathogens responsible of ocular fungal infections and showed that voriconazole was effective against most pathogens including mold and concluded that voriconazole could be used in ocular infections. In another study that investigated the reliability of intravitreal voriconazole, researchers showed that there was no retinal toxicity with doses ≤25 μg/mL in rats and argued that 100 μg voriconazole can be used for human vitreous body of 4 ml [7]. We administered amphotericin-B (3 mg/kg/day) IV and into the anterior chamber (5 μg/0.1 cc) empirically. As soon as isolating *Scopulariopsis *spp. from the culture, antifungal susceptibility testing was carried out. As a result MICs for amphotericin-B, voriconazole and caspofungin were >32 μg/mL, 8 μg/mL and 4 μg/mL, respectively. Considering its high MIC value, 2.5 μg/0.1 cc voriconazole was administered into the vitreous filled with silicon. Concurrently, amphotericin-B was replaced by IV voriconazole (4 mg/kg, b.i.d) and caspofungin (Loading dose on day 1: 70 mg, Maintenance dose: 50 mg/day). Following this treatment, clinical improvement was achieved in the patient. However, on day 10 of the treatment hepatic enzymes became elevated and this was accepted as toxic hepatitis. IV voriconazole was stopped and hepatic enzymes returned to normal during follow-up. Treatment was continued with IV caspofungin and locally administered voriconazole solution for 8 weeks.

There are few cases successfully treated with combination antifungal therapy in the literature [9,10]. Similarly, in our patients we used combination antifungal therapy. Unfortunately, one of our patient experienced side effects due to voriconazole and the other to amphotericin-B. Treatments were continued with caspofungin.

In conclusion, the success was not totally related to treatment with caspofungin. Local treatment with an antifungal agent and even a short course of another systemic antifungal also contributed to the good outcome. Both local and systemic antifungal therapy may be the best approach in endophthalmitis caused by resistant fungi.

## Competing interests

The author(s) declare that they have no competing interests.

## Authors' contributions

SA participated in the operation of both patients and helped to draft the manuscript. BE participated in manuscript design and coordination and involved in drafting the manuscript. He also prescribed the antifungal therapy of both patients. BG carried out the microbiologic studies and helped to draft the manuscript. GU followed the clinical improvement of both patients the hospitalization period. EK participated in the operation of both patients and helped to draft the manuscript. All of the authors have given final approval of the version to be published

## Pre-publication history

The pre-publication history for this paper can be accessed here:


